# A multilevel analysis on the relationship between neighbourhood poverty and public hospital utilization: is the high Indigenous morbidity avoidable?

**DOI:** 10.1186/1471-2458-11-737

**Published:** 2011-09-27

**Authors:** Yuejen Zhao, Jiqiong You, Steven L Guthridge, Andy H Lee

**Affiliations:** 1 Health Gains Planning Branch, Northern Territory Department of Health, PO Box 40596, Casuarina NT 0811, Australia; 2 School of Public Health, Curtin University, GPO Box U1987, Perth WA 6845, Australia

## Abstract

**Background:**

The estimated life expectancy at birth for Indigenous Australians is 10-11 years less than the general Australian population. The mean family income for Indigenous people is also significantly lower than for non-Indigenous people. In this paper we examine poverty or socioeconomic disadvantage as an explanation for the Indigenous health gap in hospital morbidity in Australia.

**Methods:**

We utilised a cross-sectional and ecological design using the Northern Territory public hospitalisation data from 1 July 2004 to 30 June 2008 and socio-economic indexes for areas (SEIFA) from the 2006 census. Multilevel logistic regression models were used to estimate odds ratios and confidence intervals. Both total and potentially avoidable hospitalisations were investigated.

**Results:**

This study indicated that lifting SEIFA scores for family income and education/occupation by two quintile categories for low socio-economic Indigenous groups was sufficient to overcome the excess hospital utilisation among the Indigenous population compared with the non-Indigenous population. The results support a reframing of the Indigenous health gap as being a consequence of poverty and not simplistically of ethnicity.

**Conclusions:**

Socio-economic disadvantage is a likely explanation for a substantial proportion of the hospital morbidity gap between Indigenous and non-Indigenous populations. Efforts to improve Indigenous health outcomes should recognise poverty as an underlying determinant of the health gap.

## Background

Poverty is an unacceptable human condition characterised by sustained deprivation of resources, capabilities, choices, security and power, which denies people an adequate standard of living and other human rights [1,2]. Poverty is multi-faceted, encompassing not only income and consumption, but also economic well-being and social inclusion. Socio-economic status (SES) links poverty with social, economic, occupational and educational aspects of the equation [1]. Globally one in four people live in absolute poverty and one in three people live in relative poverty [3]. In relation to health, the empirical evidence is overwhelming that the poor tend to die earlier and have higher levels of morbidity than the better-off [4]. Low SES affects health adversely throughout the life course and between generations [5,6]. Low SES is often compounded by ill-health conditions, for example, infectious diseases, perinatal conditions, diabetes, cancers, cardiovascular diseases and injuries [7-11]. This strong association does not equate to causality. Evidence suggests that causality between poverty and poor health runs in both directions: poverty generates ill-health, ill-health exacerbates poverty [4]. It is intuitive to believe that low SES can affect health both directly (low income, poor nutrition, overcrowding, poor sanitation and stress) and indirectly (loss of power, poor self-esteem and loss of access to resources), and ill-health in turn harms earning ability, employment opportunity and economic prospects, causing deeper poverty.

Between 2005 and 2007 the estimated life expectancy at birth for Indigenous Australians was 9.5-11.3 years less than the general Australian population [12]. Several researchers highlighted that among similar developed countries, Australia had the widest life expectancy gap between Indigenous and non-Indigenous populations, and was the only country in which this gap was widening [13]. Indigenous health inequality is also apparent in the disparate levels of disability and morbidity measured in public hospital admission rates [14,15]. The Australian Government has stated its determination to close the health gap between the Indigenous and non-Indigenous populations within a generation, and halve the education and employment gap within a decade [16].

There was growing evidence that poverty was responsible for poor Indigenous health [17,18]. Colonisation, loss of land, social exclusion and poverty had detrimental effects on the health of Indigenous people [17]. In Australia, Indigenous disadvantage has been identified as a key element of poverty. Between one third and one half of the Indigenous health gap could be attributed to differences in SES [19]. The Indigenous health gap may in fact not be an Indigenous issue per se, but a health issue driven by poverty. This argument has particular pertinence to the Northern Territory (NT) where approximately 30% of the total population are Indigenous, 70% of the Indigenous population live in low socio-economic areas and the public hospital care is provided to the residents free of charge.

Through determining the correlation between poverty and health service utilisation, this study aims to illuminate the issue whether it is possible to bridge the health gap by eradicating poverty for Indigenous people. The findings will provide insight into the genesis and nature of the Indigenous health gap and inform strategies to close that gap.

## Methods

### Data and setting

Hospital admissions between 1 July 2004 and 30 June 2008 were drawn from the NT public hospital morbidity database collated by the NT Department of Health (DOH). There are five public hospitals in the NT: Royal Darwin Hospital, Alice Springs Hospital, Katherine Hospital, Gove District Hospital and Tennant Creek Hospital. There is only one private hospital in the NT (Darwin Private Hospital). The public hospitals provide most of acute care services in the NT. The use of public hospital data and the selection of study period were mainly driven by data availability. The public hospitalisation data were analysed by personal demographic categories: age group, sex, Indigenous status and statistical local area (SLA) for NT residents. SLA is a general purpose spatial unit used by Australian Bureau of Statistics (ABS). There were 96 SLAs in the NT in 2006. At the patient level, the demographics were gathered during hospital admission. Annual average number of hospital inpatients per 1000 population (hereafter called hospital morbidity rate) was used as a key indicator for hospital utilisation. The reason for using number of inpatients rather than number of admissions was to minimise the bias caused by high frequency hospitalisations (for example, haemodialysis) and hospital deaths. Age was based on the first admission. Potentially avoidable hospitalisations are identified in accordance with the lists of conditions produced by the University of Adelaide [20], using diagnosis and procedure codes of International Statistical Classification and Related Health Problems, 10th Revision, Australian Modification. In Australia, population level SES is measured by socio-economic indexes for areas (SEIFA) [21]. The patient level SLAs were determined using 1250 residential locality codes and linked with SLA level SEIFA scores.

The 2006 SLA level aggregate SEIFA data were downloaded from the ABS website [21]. The SEIFA scores were national rankings derived from 35 socio-economic variables collected during the 2006 census including income, education, employment, occupation, housing and other indicators of relative advantage and disadvantage [21]. There were four types of SEIFA scores, which were constructed to reflect different aspects of SES. SEIFA1 (index of relative socio-economic disadvantage) included Indigenous status in its composition. Therefore, it was dismissed due to potential collinearity with the covariate Indigenous status used in this study. Consequently, SEIFA1 was only listed for completeness and served as a reference. SEIFA2 (index of relative socio-economic advantage and disadvantage), SEIFA3 (index of economic resources) and SEIFA4 (index of education and occupation) were applied to indicate general SES, income and education/occupation, respectively. For all of these indexes, a higher score indicates a better average SES for the area. The original SEIFA distribution for the NT appeared U-shaped because of a lack of mid-level scores. New deciles were recalculated from the original SEIFA scores. The new NT SEIFA scores were uniformly distributed between 1 and 100. This transformation was necessary to improve robustness, stability and interpretability of modelling results and to reduce confounding of the parameter estimates. There were 90 NT SLAs with valid SEIFA scores. Hospitalisations of interstate residents (7.3%) or with an unknown SLA (0.2%) were removed from the analysis. The 2006 NT resident population estimates by age group, sex and SLA were sourced from the ABS and used as the midpoint denominator. Indigenous populations were derived using the ABS experimental estimates [22].

### Multilevel modelling

A bubble diagram was first applied to examine SLA level ecologic associations between hospital morbidity rate and SEIFA scores, with the bubble size indicating population. The Pearson coefficient was used to measure the SLA level correlation. However, this SLA level correlation may overestimate the true association at the individual level and likely suffers from ecologic fallacy [23], which can be addressed by adopting a multilevel modelling approach [24]. A multilevel logistic model was chosen to investigate the connections between aggregate SEIFA scores and individual hospital utilisation, taking into account the hierarchical structure of random effects (to avoid ecologic fallacy). Level 1 referred to individual inpatients and level 2 to SLA. Individual inpatients were nested within the SLAs. The two-level model is in the form of

$$\left\{\begin{array}{c} logit\left(P_{ij}\right)=\alpha_{0j}+\alpha_{1j}\cdot Ind_{ij}+\alpha_{2j}\cdot Aged_{ij}+\alpha_{3j}\cdot Sex_{ij}+\varepsilon_{ij}\\ \alpha_{0j}=\beta_{00}+\nu_{0j}\\ \alpha_{1j}=\beta_{10}+\beta_{11}\cdot SEIFA_{j}+\nu_{1j}\\ \alpha_{2j}=\beta_{20}+\beta_{21}\cdot SEIFA_{j}+\nu_{2j} \end{array}\right)$$

where patient *i *= 1, ..., *n_j_*, *j *= 1, ..., number of SLAs, and *P_ij _*is the probability of being hospitalised during the study period. The customary link function is logit, and the corresponding probability distribution is binomial. Both total and potentially avoidable hospital morbidity were examined. Three dichotomised demographic variables were included, namely, *Ind_ij _*Indigenous status (0 = non-Indigenous, 1 = Indigenous), *Aged_ij _*aged over 50 years at first admission (0 = no, 1 = yes), and *Sex_ij _*sex (0 = male, 1 = female). Age was chosen as a dichotomous variable rather than a continuous variable, because the relationship between morbidity and age is not monotonic. The cut-off point of 50 years for *Aged *was chosen to indicate if the inpatient was an aged person [25], because 50 was close to the median age at death (52 years) for the NT and the Indigenous life expectancy at birth was about 16-20 years shorter than the non-Indigenous population in the NT [26]. The new SEIFA scores were coded -2, -1, 0, 1 and 2 from the lowest to the highest quintile as an ordinal index. The regression coefficients *α *and *β *of a particular variable pertains to the risk of hospitalisation. All level 1 predictors, except sex, exhibited random effects.

Three models were designed. Models 1 and 2 were devised to test the general SES with **SEIFA***j *using SEIFA1 and SEIFA2 respectively. In Model 3, family income and education were tested simultaneously by applying SEIFA3 and SEIFA4 in the same model. For simplicity, no interaction terms were imposed between SES, Indigenous status and age groups in the models. This assumption was reasonable because the relation between health and SES can be expected to be consistent between Indigenous and non-Indigenous people and consistent across age groups. Multilevel logistic regression modelling was undertaken using the GLLAMM procedure in the Stata package release 11. Standardised residuals and concordance coefficients (ρ_c_) between observed and fitted values for the hospital morbidity rate were used to assess goodness-of-fit of the models [27,28].

### Ethics approval

The study protocol was approved by the Human Research Ethics Committee of DOH and Menzies School of Health Research (approval number HREC-2010-1373).

## Results

**Figure 1:**
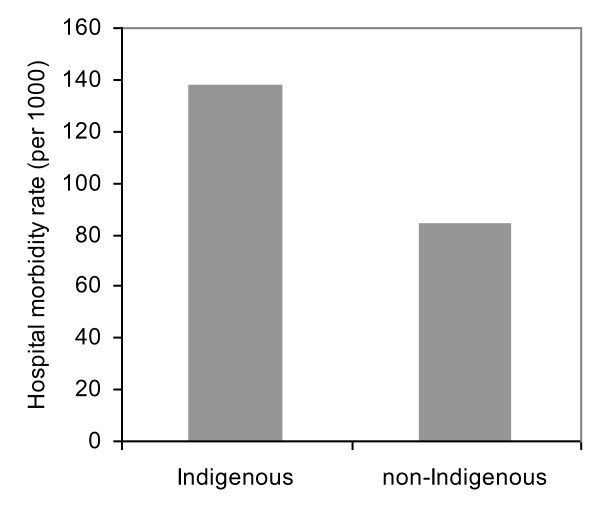
**Annual average number of hospital inpatients per 1000 population by Indigenous status, Northern Territory, Australia, 2004/5-2007/8**.

**Figure 2:**
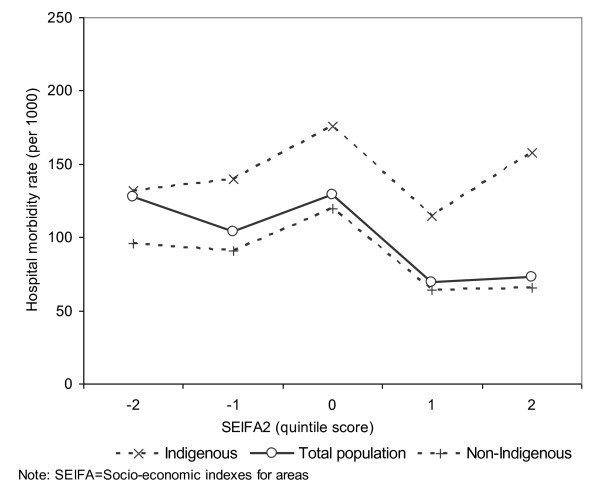
**Socioeconomic index for areas and annual average number of hospital inpatients per 1000 population by Indigenous status, Northern Territory, Australia, 2004/5-2007/8**.

A total of 345, 040 hospitalisations from 1 July 2004 to 30 June 2008 were analysed. The resulting number of hospital inpatients was 84, 729 during the four-year period, 54% of whom were female and 42% Indigenous. This compared with the NT population proportions of 48% female and 30% Indigenous. Figure 1 highlights that the overall hospital morbidity in NT Indigenous population was over 60% higher than that of non-Indigenous. Figure 2 reveals that there appeared to be a negative gradient between the overall hospital morbidity rates and SEIFA2 quintiles (see solid line in Figure 2). Additionally, because more Indigenous people live in low SES remote areas and more non Indigenous people in high SES urban areas, the high hospital morbidity in the lowest quintile was mainly driven by Indigenous morbidity, and the reverse is generally true for non-Indigenous population (see dashed lines in Figure 2). Indigenous morbidity appears higher than its non-Indigenous counterpart for all SEIFA2 quintiles. The major public hospitals are located in high SES areas, and migration of inpatients for easy access to public hospitals [29] may explain the high Indigenous morbidity in the highest SEIFA2 quintiles.

**Figure 3:**
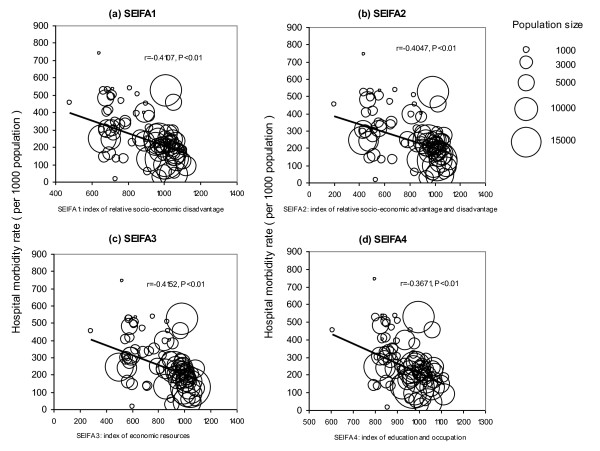
**Correlations between Socio-economic Indexes for Areas (SEIFA) scores and annual average numbers of hospital inpatients per 1000 population for the statistical local areas, Northern Territory, Australia, 2004/5-2007/8**.

Figure 3 shows that the four raw SEIFA scores were negatively correlated with the annual hospital morbidity rate by SLA. The Pearson coefficients (r) ranged from -0.4107 for SEIFA1 to -0.3671 for SEIFA4 (P < 0.01). It was evident that at SLA level, the lower the average SEIFA scores (being worse-off), the higher the hospital morbidity rate.

The coefficients, standard errors (SE), odds ratios (OR) and their 95% confidence intervals (CI) for four multilevel models (three for total hospital morbidity and one for potentially avoidable hospital morbidity) are compared in Table 1. The coefficients of SEIFA scores were all negative, suggesting that a higher hospital morbidity rate was associated with a lower level of SES. Model 1 was dismissed due to potential interdependence. In accord with the log likelihood, Model 3a (the income and education model) was preferred over Models 1 and 2. All ORs were statistically significant. The adjusted ORs for the income and education model suggested that being Indigenous was associated with increased odds (29.7%) of hospitalisation. Being female or aged 50 years and over also markedly increased the risk of hospitalisation by 36.6% and 22.3% respectively. By contrast, a high SEIFA score was related to a decreased chance of hospitalisation. From Model 1 to Models 2 and 3a, the association between Indigenous status and morbidity has been attenuated and the ORs decreased from 1.818 to 1.297. The corresponding ORs for SEIFA scores have changed from 0.960 to 0.926 and 0.917, suggesting that high SES tended to reduce the hospitalisation risk by 4.0-8.3% if the SEIFA scores were modified up by a quintile category. In Model 2, changing SEIFA2 from the lowest quintile to the highest (4 × 0.079 = 0.316) would nearly offset the Indigenous difference (0.342). In Model 3a when family income and education are jointly considered, changing SEIFA3 and SEIFA4 simultaneously four times from the lowest to the highest quintile (4 × 0.077+4 × 0.087 = 0.656) far offsets the Indigenous difference (0.260). Changing one quintile category for family income and education would lead respectively to a 7.4% and 8.3% reduction in the risk of hospitalisations on the multiplicative scale. Considering avoidable hospital morbidity in Model 3b yielded similar results which reinforced the point that improvements in family income and education levels would significantly offset the high risk of hospitalisation for Indigenous people. The variance of random effects showed significant between-SLA contextual variations in hospital morbidity rates.

**Table 1:** Multilevel logistic models for number of total and potentially avoidable hospital patients per population

	(1) Disadvantage model				(2) Advantage and disadvantage model			
		
Coefficient	*β*	(SE)	OR	(95%CI)	*β*	(SE)	OR	(95%CI)
Intercept	-1.963	(0.008)			-1.761	(0.008)		
Indigenous status	0.598	(0.011)	1.818	(1.777-1.860)	0.342	(0.012)	1.408	(1.375-1.441)
Sex	0.308	(0.008)	1.361	(1.339-1.383)	0.311	(0.008)	1.365	(1.343-1.388)
Aged	0.144	(0.012)	1.155	(1.127-1.184)	0.138	(0.013)	1.148	(1.118-1.179)
SEIFA 1	-0.041	(0.004)	0.960	(0.953-0.967)				
SEIFA 2					-0.079	(0.004)	0.924	(0.917-0.931)
-Log likelihood	185142				185246			
Variance of random effect								
Intercept	0.503	(0.007)			0.476	(0.007)		
Indigenous status	0.393	(0.012)			0.345	(0.012)		
Aged	0.082	(0.006)			0.076	(0.006)		

	**(3) Income and education model**							
	
	**a. Total hospital morbidity**				**b. Avoidable hospital morbidity**			
		
Intercept	-1.811	(0.008)			-2.378	(0.012)		
Indigenous status	0.260	(0.013)	1.297	(1.265-1.329)	0.529	(0.015)	1.697	(1.646-1.748)
Sex	0.312	(0.008)	1.366	(1.344-1.389)	0.270	(0.010)	1.310	(1.284-1.336)
Aged	0.202	(0.013)	1.223	(1.192-1.256)	0.003	(0.000)	1.003	(1.002-1.003)
SEIFA 3	-0.077	(0.004)	0.926	(0.918-0.934)	-0.045	(0.006)	0.956	(0.945-0.967)
SEIFA 4	-0.087	(0.004)	0.917	(0.909-0.925)	-0.139	(0.005)	0.870	(0.861-0.879)
-Log likelihood	185076				138337			
Variance of random effect								
Intercept	0.556	(0.009)			0.336	(0.009)		
Indigenous status	0.399	(0.013)			0.200	(0.009)		
Aged	0.042	(0.003)			0.000	(0.000)		

Note: ***β ***= coefficient estimate; CI = confidence interval; OR = Odds Ratio; SE = Standard error; SEIFA = Socio-Economic Indexes for Areas; SEIFA1 measures relative socio-economic disadvantage; SEIFA2 represents relative socio-economic advantage and disadvantage; SEIFA3 indicates household income; SEIFA4 is index of education; SLA = Statistical Local Area.

Finally, sensitivity analysis showed a close concordance (ρ_c _= 0.989, P < 0.01) between the observed and expected values of the hospital morbidity rates for the optimal income and education model (Model 3a), and standardised residuals did not identify any apparent lack of fit. Changing the age cut-off for an aged person to 52 years (median age at death) and 60 years caused little change in the results (not shown for brevity).

## Discussion

The association between SES and hospital morbidity patterns indicates that low SES may explain a substantial proportion of the health gap between Indigenous and non-Indigenous populations. Our analysis indicated that simultaneously lifting family income and education/occupation for low SES Indigenous groups by two quintile categories could significantly offset the difference in hospital morbidity between Indigenous and non-Indigenous populations. The results are important as they reframe the health gap from a non-modifiable factor based on ethnicity to a modifiable determinant of social disadvantage. The results emphasise the importance of addressing social inequality to closing the health gap.

There is strong evidence that changing socioeconomic disadvantage can significantly improve Indigenous health [17,30-32]. Poverty or low SES is a modifiable risk factor which has been widely associated with high mortality and morbidity [33,34]. Our findings are consistent with the argument that small changes in socio-economic circumstances can significantly influence health outcomes [30]. They are also consistent with international and Australian studies [35,36], which have proposed that socio-economic inequalities may be behind Indigenous health gap. Previous research suggested that SLA level SEIFA data provide a reliable indication of socio-economic disadvantage for areas, although they may understate Indigenous disadvantage [37]. The specific mechanisms responsible for the associations of SES disadvantage with increased risk of hospitalisation remain to be explored. By contrast, genetic factors have been reported as having only a limited role in relation to Indigenous health differentials [38]. There were at least three pathways to link SES disadvantage to poor health: individual access to resources, social capital and individual psychosocial perception [39]. In 2006, the mean equivalised gross household income for Indigenous people was $460 per week, compared with $740 for non-Indigenous people [40]. Although diseases directly linked to poor living conditions and environment such as tuberculosis and pneumonia are relatively uncommon in Australia [41], they are more common among low SES population subgroups including Indigenous people. Chronic diseases, including cardiovascular disease, diabetes and cancers, have emerged as a new challenge for low SES population. Community environments may be a fourth pathway through which social structure influences health, with many health risks such as tobacco smoking, alcohol use, child maltreatment all being socio-economically patterned. Remoteness also affects both SES and health [42]. Mean equivalised household income was lower in remote areas compared with non-remote areas for Indigenous people ($329 in very remote areas and $539 per week in major cities). Remoteness is highly correlated with SES in the NT and therefore not included in the models due to collinearity.

The advantage of using multilevel modelling is that it takes the hierarchical structure of the data into account by specifying random effects at each level of analysis. Compared with the standard (single-level) logistic model, a multilevel model produces more accurate parameter estimates with realistic confidence intervals. The interpretation of the ORs is also different. Multilevel ORs represent individual change in response probability affected by the predictor within each SLA, whereas ordinary ORs represent the average change in response probability affected by the predictor across all SLAs. Assessment of exposures at a community level in a social context may lead to a better understanding of social determinants of health that is more than the sum of individual measures [30]. By analysing and integrating both contextual and individual level variables, multilevel modelling enables research into the social and collective determinants of health. This study found that the SLA has an independent effect on the risk for poor health, adjusting for certain individual characteristics. The contextual effects identified provide evidence for community-based interventions. This study has advanced our knowledge about why the Indigenous population are subject to higher hospital morbidity. Applied appropriately in socioeconomic studies in health, the multilevel modelling is useful for quantifying individual level associations using grouped data.

There were also several possible limitations. SEIFA is an aggregate SES measure for the geographic areas. Although SEIFA has been widely applied in social, economic and public health research [33,43], in this study SEIFA was applied at an individual level and the results must therefore only be regarded as indicative. This limitation could be addressed in future by collecting and analysing individual level SES information and linking with hospitalisation data. SEIFA scores are relative as changing them for one group would affect the relative scores for the whole population. Nonetheless, in this study the changing of SEIFA scores was used to indicate improvements in SES. This report focused on hospital inpatient data. The health gaps in terms of mortality and life expectancy have been described elsewhere [44]. The hospital morbidity rates in this study arguably underrepresented the health care needs by Indigenous people because of differentials associated with poor access to hospital services [37,45]. This study focused on the global impact of poverty on hospitalisations, and not on the causality of hospital morbidity. The high correlations of hospital morbidity and mortality with SES measures have been demonstrated previously in Australia by using area based data [46]. The cross-sectional design of the study can only demonstrate an association between factors and randomised experiments are needed to accurately assess the causality between poverty and ill-health [47]. Further research on primary care avoidable hospitalisations by condition is desirable. There were no complete costing data and primary care access data available for this study period. Finally, as indicated in Figure 3, the majority of variation in morbidity is not explained by SEIFA. There are many variables not included in this model, which could potentially explain both SIEFA and differential health.

Taking action on the social determinants of health is never simple and international experience in poverty alleviation has demonstrated the complexity of addressing social determinants of health [48]. Indigenous health inequalities were precipitated by historical factors unique to Indigenous people including colonisation, loss of language and culture [17]. Action to redress the inequality must go beyond the traditional approach of health care and focus on the root causes, including poverty. Eliminating poverty must be through public policy and action. Capacity building and sustained economic development may hold the key to close the Indigenous health gap.

## Conclusions

This study linked the Indigenous health gap with socio-economic disadvantage, using hospital morbidity information and multilevel logistic regression models. The statistical analyses of hospital morbidity based on socioeconomic measures show a complex pattern and point to the importance of including poverty alleviation in health policy for the Indigenous population, especially in rural and remote areas. Improved outcomes in poverty eradication and education program for Indigenous people will ease the pressure on the public health system and in the long term, reduce government health spending. Efforts to address Indigenous health issues should recognise poverty as an underlying factor of the health gap. Without eliminating poverty, efforts to reduce the Indigenous health gap will be ineffective and inefficient.

## Competing interests

The authors declare that they have no competing interests.

## Authors' contributions

All authors contributed to the design of the study. YZ, JQ and AHL contributed to data collection and statistical analysis. SLG led the writing and revision of the paper. All authors read and approved the final manuscript.

## Pre-publication history

The pre-publication history for this paper can be accessed here:

http://www.biomedcentral.com/1471-2458/11/737/prepub
